# Revitalizing medical schools in late sixteenth-century China: Lü Kun and the medical reform program in his *Shizheng Lu*

**DOI:** 10.5195/jmla.2024.1732

**Published:** 2024-04-01

**Authors:** Jiao Kun

**Affiliations:** 1 jkjiao@whu.edu.cn, Associate Professor, School of History, Wuhan University, Wuhan, China.

**Keywords:** Lü Kun, Shizheng Lu, Ming China, Medical School, Public Health System

## Abstract

This article takes a glance at the medial reform program recorded in the book *Shizheng Lu* 實政錄 (Records of Practical Policies for Governing) by Lü Kun, a scholar-official from Ming China who was active more than 400 years ago. The *Shizheng Lu* is a compilation of varied policies and plans designed by Lü Kun as a local official to restore and improve administration of civic affairs. A sub-chapter in this book is devoted to the subject of public health service. Analysis of this text yields knowledge of how the local public health system in Ming China was supposed to operate, pivoting on the key role of medical schools and highlighting the severe malfunction of this system in Lü Kun's time. The same text also sheds light on a handful of popular medical books from the era that could have been used for medical education.

## INTRODUCTION

The history of China is rich with the development of institutions for centralized administration of public affairs, of which medical care for the people has constituted an important branch. A nationwide public health system designed and run by the state can be traced back at least to the Northern Song period (AD 960–1127). Traits of this system were inherited or replaced with new ones by successive dynasties and governments, but the general practice of centralized medical administration has continued through almost all the historical periods thereafter into the current time.

In recent years, the public health care and medical administration system in pre-modern China has received growing attention from historians of China, especially those interested in traditional Chinese society. However, despite the plentiful and insightful works so far by scholars from both within and outside of China, in light of the lengthy span of the tradition of centralized medical administration in China, as well as rather sporadic and scattered records pertaining to this subject, much remains to be explored and discussed in this field of research.

The Ming dynasty (AD 1368–1644) existed for nearly 300 years and constituted an important phase of late imperial China. Its medical care system is hence rightfully worthy of observation and discussion. Nonetheless, people sometimes find the Ming to be a weak link in the study of Chinese medical history, compared both to the preceding two Songs and the following Qing. As for Ming China's medical administration at the local level, our understanding is even more vague. Fortunately, a scholar-official named Lü Kun from the late sixteenth century left future generations with a book recording his policies in various aspects as a local official and providing a glimpse into how public health affairs were managed in localities of China about 400 years ago.

## ABOUT LÜ KUN AND THE BOOK *SHIZHENG LU*

Lü Kun 呂坤, from Ningling 寧陵 County, Henan 河南 Province (the same county and province still exist today), was born in 1536, or the fifteenth year of the reign of Emperor Jiajing 嘉靖, the eleventh monarch of the Ming dynasty. In 1574, Lü Kun passed the highest level of the civil service examinations to get his *jinshi* 進士 (metropolitan graduate) degree, immediately allowing him to become an official of the empire. Lü started his political career as magistrate of different counties and then served in a series of positions, ranging from mid-rank court official to provincial top. After twenty years of service, Lü was promoted to a high-ranking position at court and summoned to the capital, Beijing. However, in Beijing, he soon got entangled in factional struggles within the court of Emperor Wanli 萬曆 and was forced to retire in 1597. Lü Kun spent the rest of his life in his hometown until his death in 1618, never returning to officialdom.

Lü Kun both launched and finished his political career under the reign of Emperor Wanli, who ruled for forty-eight years, only to see the Ming dynasty traveling along the trajectory of decline and eventually onto the precipice of collapse. Social, political, and military crises kept mounting. An expanding population consumed all the extra products made available through economic growth, which further drove impoverished people into vagrancy as well as anti-government religious sects. Mongols and Jurchens encroached on the northern frontier, and there was increasing military pressure from a reunified Japan. Finally, fierce factional disputes over policies and personnel arrangements within the court almost paralyzed the Ming high politics.

As pointed out by Joanna F. Handlin, Lü Kun like many of his contemporary politicians shared in the then-prevailing strong sense of crisis [1]. In addition, his posts as a local official were all in the north, a much poorer land with an inadequately maintained social infrastructure compared to the wealthy southeastern part of China. All these factors added up to Lü Kun's clear proclivity for statecraft, as embodied in the book *Shizheng Lu* and many other of his works.

According to Xie Yang [2], the *Shizheng Lu* was initially compiled and published not by Lü Kun himself, but by a certain Zhao Wenbing 趙文炳 who claimed to be Lü's student. Zhao first collected stand-alone works of Lü Kun and compiled them into a seven-chapter version of the *Shizheng Lu*; soon after the publication of this version, he added another two chapters and republished the book. About twenty years later, a local official named Fu Shuxun 傅淑訓 compiled and published a ten-chapter version. The above-mentioned three versions are extant today, but the earliest seven-chapter version was the most popular one until recently, while the rarest ten-chapter version leaves only one copy today in its original form, which is now kept at the library of Sun Yat-sen University, China.

**Figure 1:**
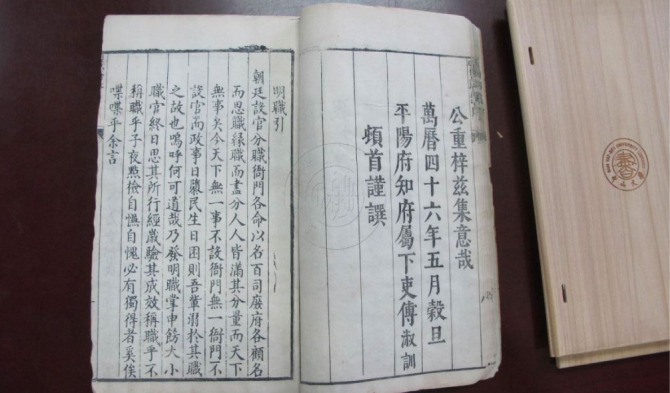
Two pages from the sole extant copy of the ten-chapter version *Shizheng Lu*. The page on the right, which marks the end of a preface by Fu Shuxun, carries the date of the fifth month of the 46th year of the Wanli reign (June or July 1618), the year Lü Kun died. Courtesy of the library of Sun Yat-sen University.

During the following Qing dynasty, the *Shizheng Lu* gained popularity among officials and was frequently reprinted, sometimes with adaptation, or it was incorporated into different versions of collected works of Lü Kun. In 2008, a revised and punctuated version of the *Lü Kun Quanji* 呂坤全集 (Complete Works of Lü Kun) was published by Zhonghua shuju 中華書局 in Beijing. It should be regarded as the most reliable and accessible edition of Lü Kun's works for today's readers. Of course, it contains the *Shizheng Lu*, the text of which is in essence based on the nine-chapter version by Zhao Wenbing. Considering that the ten-chapter version is in fact an abridged nine-chapter version with only one additional chapter, the following discussion will be based on the text in *Lü Kun Quanji.*

The *Shizheng Lu* consists of policies as well as reform plans devised and implemented by Lü Kun in different periods, all of which are compiled as chapters pertaining to a certain area of civic administration or organization of local government. In the second chapter titled “Minwu 民務 (Civic Affairs),” which focuses on supporting the livelihoods of commoners, Lü Kun set a sub-chapter titled “Zhenju Yixue 振舉醫學 (Revitalizing the Medical Schools).” This sub-chapter provides us with key information about local medical administration in Lü Kun's time.

**Figure 2:**
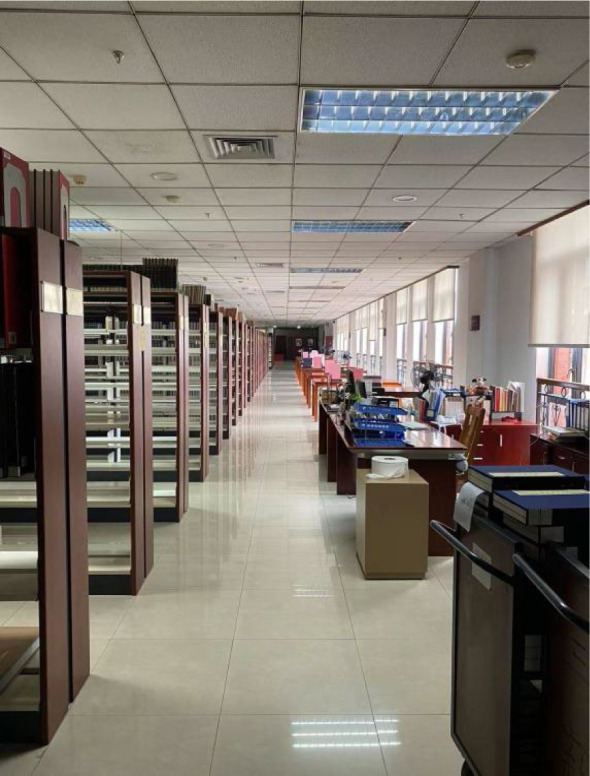
A View of the ancient book section of the library of Sun Yat-sen University, which possesses a vast collection of books published in China before 1912, including the ten-chapter version *Shizheng Lu*. Photographed and provided generously by Huang Youhao, PhD Candidate at the Department of History, Sun Yat-sen University.

## LOCAL MEDICAL ADMINISTRATION IN MING CHINA

The chapter “Minwu” in the *Shizheng lu* is believed to have been originally written by Lü Kun between 1592 and 1593 [3], while he served as the Grand Coordinator of Shanxi Province, a post powerful enough to supervise all the civic affairs in that province. The sub-chapter “Zhenju Yixue” includes a short introduction and sixteen specific orders or instructions issued by Lü Kun, aiming to restore local medical administration and improve public health service to the ordinary people. What first strikes as shocking when reading these paragraphs, however, is the miserable picture of declined or even decayed local public health service depicted by Lü Kun. He deplored that “the buildings of medical schools have remained collapsed or even been sold out” [4]; “the officials cared so little about medical affairs” [5] as to leave town residents without the most fundamental medical knowledge in the post of medical officials; and quacks who could not even read were killing numerous patients every year. Lü Kun blamed the malfunction of the local public health system on the neglect of duty and indolence by local officials, and he introduced a series of polices trying to restore medical administration in the localities, centered on the goal to “revitalize the medical schools.”

**Figure 3:**
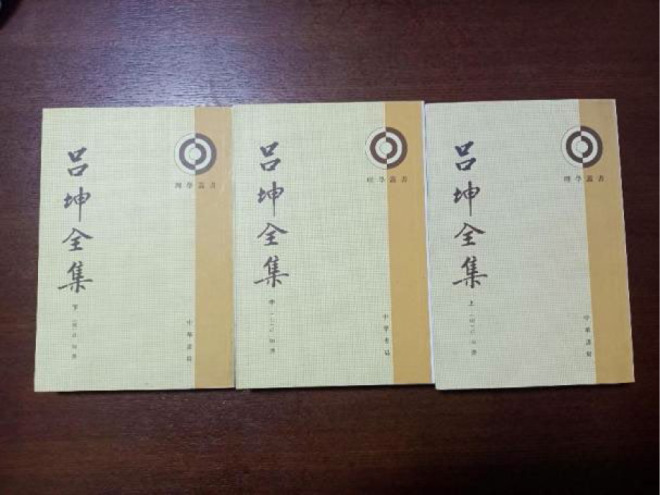
Front covers of the three-volume *Lü Kun Quanji* published in 2008 by Zhonghua shuju, Beijing. Photographed by the author.

China's astonishingly long history of state-run medical institutions allegedly dates back to the Western Zhou (1046–771 BC) period, and medical schools set up by the court are said to have first appeared as early as the year 443 CE [6]. In the Tang (AD 618–907), Song (AD 960–1279), and Yuan (AD 1271–1368) dynasties, medical schools run by the government were expanded to localities. The Ming dynasty inherited this trend and ordered shortly after its foundation the establishment of a medical school in every county, sub-prefecture, and prefecture [7]. From records in the sub-chapter “Zhenju yixue,” it is clear that Ming medical schools functioned not just as educational institutions but actually as the hub of the local public health system: the principals of medical schools were at the same time local medical officials; the schools sent teachers and students out to treat patients, making themselves public hospitals; and they also prepared and disbursed medications as public pharmacies. This is the reason that Lü Kun viewed restoring medical schools as the keystone to his reform plan for medical administration.

## EFFORTS TO REVITALIZE THE MEDICAL SCHOOLS

In the sixteen carefully crafted policies listed in the sub-chapter “Zhenju yixue,” Lü Kun offered a series of specific instructions to local officials under his jurisdiction on managing public health affairs. He ordered these officials to repair and rebuild medical schools; put conscientious, reputable doctors in charge of them as medical officials; and make sure medical schools had enough funding to operate. He also made it clear that medical officials should purchase medical materials personally every month and supervise students preparing medications. Local officials and their family members were forbidden to embezzle medications from the charity pharmacy (Huimin Yaoju 惠民藥局), which was charged with handing out medicines to the poor for free. The charity pharmacy as a branch of the public health system had a long history that dated back to the Northern Song [8]; entering the Ming period it became a facility affiliated with the medical school.

Along with these instructions, Lü Kun also put great emphasis on improving the education of students at medical schools and then sending them out to treat patients once they achieved a certain level of professionalism. To our great convenience and of great value, Lü Kun meticulously recorded the titles of two dozen medical books he considered suitable for use as textbooks at the medical schools. These books range from ancient texts deemed classics of traditional Chinese medical theory, such as *Suwen* 素問 (Plain Questions) and *Lingshu* 靈樞 (Spiritual Pivot), to works on materia medica, medical formulas, books of prescriptions, and comprehensive medical books from the Song, Yuan, and Ming periods. The full list includes: *Suwen*, *Lingshu*, *Dongyuan Shi Shu* 東垣十書 (Ten Books of Dongyuan), *Yixue Gangmu* 醫學綱目 (Detailed Outline of Medicine), *Yixue Rumen* 醫學入門 (Introduction to Medicine), *Yilin Jiyao* 醫林集要 (Essentials of Medical Knowledge), *Yijing Xiaoxue* 醫經小學 (Primer on Medical Classics), *Yixue Zhengchuan* 醫學正傳 (Correct Teachings about Medicine), *Yuji Weiyi* 玉機微義 (Profound Teachings of the Big Dipper), *Renzhai Zhizhi* 仁齋直指 (Explicit Guidance by Renzhai), *Mingyi Zhizhang* 名醫指掌 (Handbook for Renowned Doctors), *Danxi Zuanyao* 丹溪纂要 (Collected and Revised Works of Danxi), *Shanghan Liu Shu* 傷寒六書 (Six Books on Febrile Diseases), *Shanghan Zhizhang* 傷寒指掌 (Handbook for Treating Febrile Diseases), *Yifang Jiejing* 醫方捷徑 (Short Cuts to Prescriptions), *Lizhai Waike* 立齋外科 (Lizhai's External Medicine), *Douzhen Jingyan Liangfang* 痘疹經驗良方 (Empirical Excellent Medical Formulas for Pox), *Caishi Douzhen* 蔡氏痘疹 (Doctor Cai's Book about Pox), *Mingyi Fangkao* 名醫方考 (Research on Medical Formulas by Renowned Doctors), *Yifang Zhaiyao* 醫方摘要 (Summaries of Medical Formulas), *Bencao Faming* 本草發明 (Clarification on Materia Medica), *Bencao Mengquan* 本草蒙筌 (Materia Medica for Beginners), *Bencao Fahui* 本草發揮 (Elaboration on Materia Medica), and *Danxi Maijue* 丹溪脈訣 (Danxi's Pulse Formulas). Lü Kun ordered that every student pick one of these books for study under the supervision of medical officials and master the book before they were officially permitted to perform treatment as proper doctors.

## CONCLUSION

Writings on statecraft such as the *Shizheng Lu*, which also functioned as guidebooks for officials, were not rare in Ming and Qing China. Yet few among them has transmitted so richly and vividly the reality of local medical administration as Lü Kun's work. Generally, historians have seen the Ming Dynasty as marked by the state's waning interest in maintaining a costly nationwide public health system [9]. Records from the *Shizheng Lu* undoubtedly support this opinion, as they attest to the thorough malfunction of the public health system in localities. However, they also show that officials like Lü Kun considered public medical service to be an important part of the government's responsibilities and actually worked to restore it. We do not know how well Lü Kun's reform program worked, but since no existing resource tells about any conspicuous resurrection of the local public health system in late Ming, and the scale of the system shrank greatly in the following Qing period, it is at least safe to say that Lü Kun's efforts did not leave in their wake a lasting impact.

Moreover, the value of the *Shizheng Lu* lies also in preserving the titles of medical books popular and considered practical in late Ming. Lü Kun did not randomly choose the two dozen books for the teaching program at medical schools; he apparently possessed a relatively high level of medical knowledge and could even be called a medical expert in certain areas. The evidence is that among all the extant works of Lü Kun, there is a book titled *Zhenke* 疹科 (On Exanthema) that discusses the pathology and treatment of diseases including measles and chicken pox. Lü Kun also mentions in the sub-chapter “Zhenju yixue” that he was preparing to publish a book titled *Bianmin Fang* 便民方 (Medical Formulas for the People's Convenience)*,* which clearly would have collected medical formulas for common diseases in the local society. All these testify to Lü Kun's enthusiasm toward and expertise in medicine; thus, he must have picked the textbooks for medical education out of professional insight. A closer look at Lü Kun's list may shed light on interesting historical facts: for example, he took in several works on febrile diseases and pox, probably indicating the existence of endemic infectious diseases in Shanxi Province at the time. As to the medical knowledge contained in and progress in medicine represented by these books, further examination and discussion are naturally in order.
